# Neuraxial Analgesia for Scoliosis Correction: A Case Series in a Specialised Children's Centre

**DOI:** 10.7759/cureus.66344

**Published:** 2024-08-07

**Authors:** Abdulrahman A Alselaiti, Rayan Muawad, Ahmed Haroun M Mahmoud, Nezar M Alzughaibi, Ahmed ALsaad, Yasser Almashari, Ali Alneami, Saeed Abuareef

**Affiliations:** 1 Department of Pediatric Anaesthesia, King Abdulaziz Medical City, Riyadh, SAU; 2 Department of Anesthesiology, King Abdulaziz Medical City, Riyadh, SAU

**Keywords:** anaesthesia, dexmedetomidine, morphine, scoliosis, caudal analgesia

## Abstract

Background: Surgical correction of spinal deformities in children presents a challenge to the anaesthetist due to the extensive nature of the surgery, the co-morbidities of the patients and the constraints on aesthetic techniques of intraoperative neurophysiological monitoring of the spinal cord. Patients undergoing scoliosis surgery are considered to suffer severe pain, which may lead to a negative impact on patient psychology and physical well-being. By using effective postoperative pain regimens to enhance recovery after surgery, pain can be significantly reduced, leading to patient satisfaction, facilitating early mobilisation, promoting oral intake, lowering postoperative pain and shortening the length of hospital stay. Thus, the primary objectives of this study were to assess the postoperative pain management and first rescue analgesic medications, by using preservative-free morphine 50 mcg/kg and dexmedetomidine 4 mcg along with normal saline 0.5 ml kg caudally, as well as to look for the secondary objectives side effects, including respiratory depression, nausea, vomiting and pruritis, timing of postoperative ambulation and length of hospital stay.

Methods: In this study, we retrospectively included children under 14 years of age and above three years who underwent scoliosis surgery under a caudal epidural-general anaesthesia using caudal morphine and the dexmedetomidine technique in the period from January to May 2023 at the National Guard Health Affairs Hospital (NGHA), Riyadh. We collected the data of seven cases using the electronic chart system of the Best Care database to extract the specific cases that meet the inclusion criteria for the study, which are idiopathic scoliosis patients, aged 3-14 years, and primary correction procedures.

Results: The pain-free duration was between seven hours and 48 hours (about two days). There were four female cases (57.14%), and three cases were male (42.85%). The patients’ American Society of Anesthesiologists (ASA) status were II to III. In all the cases, there was no documentation of any episodes of postoperative nausea and vomiting (PONV), respiratory distress or pruritus, except for one case where the patient had an episode of PONV.

Conclusion: In this study, our aim was to present our experience with dexmedetomidine as an efficient medication when coadministered with morphine to be used in the operating room. We found a high level of reliability in prolonging analgesia time and delaying the usage of rescue medication. We encourage more studies on caudal dexmedetomidine for patients undergoing scoliosis surgery.

## Introduction

Surgical correction of spinal deformities in children presents a challenge among anaesthesiologists due to the extensive nature of the surgery, the co-morbidities of the patients and the constraints on aesthetic techniques of intraoperative neurophysiological monitoring of the spinal cord. Patients undergoing scoliosis surgery are considered to suffer severe pain, which leads to a negative impact on patient psychology and physical well-being, with the consideration that it may also involve family members. By using effective postoperative pain regimens, pain can be significantly reduced, leading to patient satisfaction, facilitating early mobilisation, promoting oral intake and shortening the length of hospital stay. Caudal analgesia plays an important role in pain management in multiple surgeries that involve thoracic, abdominal and lower limbs. Multiple studies have been done in different types of surgeries to assess postoperative pain using different medications, such as morphine and dexmedetomidine. Wang et al. (2021) found that using a dose of 1.5 mcg/kg dexmedetomidine leads to accelerating the onset of the caudal block, reducing stress and inflammation, stabilising the circulation, increasing the duration of postoperative analgesia and reducing anaesthesia and operation-associated adverse events [1]. In another study assessing caudal morphine versus intravenous morphine postoperative for patients undergoing cardiac surgery, they found that a single dose of morphine 0.06 mg/kg in caudal epidural had a significant intravenous morphine sparing effect compared to a postoperative morphine infusion and that it was associated with a low incidence of adverse events after surgery. It was also concluded that effective analgesia was achieved for 16-20 hours after surgery [2]. Generally, dexmedetomidine can deliver sedation and analgesia without respiratory depression, and one study showed interesting results on lowering the risk of emergency delirium when using dexmedetomidine 0.5 μg/kg [3]. The dexmedetomidine was mainly used within the intensive care unit, yet its usage during general anaesthesia, especially in awake fibreoptic and neurosurgical anaesthesia, was highly reported since it has been shown to play a major role in decreasing postoperative cognitive and behavioural dysfunction [4,5]. Furthermore, in a late study, dexmedetomidine usage was considered to provide antiemetic and anti-shivering effects [6]. On reviewing the literature, limited studies have analysed the efficacy of caudal anaesthesia using morphine and dexmedetomidine as postoperative strategies for pain management. This study aims to describe our experience using caudal morphine and dexmedetomidine in children undergoing scoliosis surgery.

## Materials and methods

After approval (approval number 000008224) by the Institutional Ethics Committee, King Abdullah International Medical Research Center, Riyadh and obtaining informed parental consent, we retrospectively included children under 14 years of age and above three years who underwent scoliosis surgery under combined caudal epidural-general anaesthesia using caudal morphine and the dexmedetomidine technique in the period from January to May 2023 at the National Guard Health Affairs Hospital (NGHA), Riyadh. We collected the data of seven cases using the electronic chart system of the hospital database (Best Care) to extract the specific cases that met the inclusion criteria for the study, which were idiopathic scoliosis patients, aged 3-14 years, and the primary scoliosis correction procedure. Patients with neuromuscular and cardiac disease were excluded. The data collection method was a retrospective chart review, and all data were extracted from the Best Care database in NGHA.

The characteristics of the patient were gender, age, weight, height, ASA (American Society of Anesthesiologists) status, Cobb angle, procedure duration, induction drugs and doses, and anaesthesia maintenance drugs and doses. Also, we measure the primary outcome as the duration where the patient was pain-free and the time of first rescue analgesia. Also as secondary outcomes, we assess the side effects such as nausea, vomiting, respiratory distress, pruritus, the timing of postoperative ambulation, and length of hospital stay.

The seven cases that met the inclusion criteria underwent corrective scoliosis surgery under combined caudal-general anaesthesia using caudal morphine and the dexmedetomidine technique.

Anaesthetic intervention

Preoperatively, recommended fasting times were assured, and no premedication was administered. Monitoring included pulse oximetry, electrocardiography, noninvasive and invasive arterial blood pressure, capnography and core temperature.  All patients were present at the operating room with intravenous catheters.

General anaesthesia was induced intravenously with propofol 2-3 mg/kg and fentanyl 1-2 mcg/kg boluses. No neuromuscular blockade was used. In all cases, the trachea was intubated using direct or video-assisted laryngoscopy, and general anaesthesia was maintained with oxygen and air along with propofol 180-250 mcg/kg/min, fentanyl 1-3 mcg/kg/hr and tranexamic acid 10 mcg/kg/hr infusion. The patients were positioned in lateral decubitus with their hips flexed, and the skin was disinfected with alcohol and aqueous antiseptic solution in the craniocaudal direction. Before starting the technique, the two sacral cornua were identified by palpation, and a 22-gauge cannula was used for the loss of resistance. Then, morphine 50 mcg/kg, dexmedetomidine 4 mcg and 0.5 ml/kg normal saline were injected. Throughout the surgical procedure, any drop in blood pressure by 15% of the baseline blood pressure was treated with ephedrine 0.2 mg/kg. During the procedure, every intervention and medication administered was recorded in the anaesthesia chart. After the end of surgery, all patients were extubated in the operating room and transferred to the ward or paediatric intensive care unit.  

Postoperatively, we recorded the duration for which the patients were pain-free. We also recorded any adverse events such as vomiting, pruritus and respiratory depression. The pain score was assessed using the Face, Legs, Activity, Cry, Consolability (FLACC) score. All patients were kept on regular pain medications: paracetamol 15 mg/kg every six hours, ketorolac 0.5 mg/kg every eight hours and morphine 100 mcg/kg every four hours if the FLACC score was more than 4.

## Results

Of the seven cases that met the inclusion criteria that we established in our study, there were four female cases (57.14%), while there were three male cases (42.85%) (Table 1). In this study, we encountered patients aged 8-14 years old. The BMI range was between 10.1 and 20.4. The ASA status among the patients was II and III going for posterior spinal correction with different levels. The Cobb angle degrees were different among the patients, beginning with the lowest at 51 and the highest at 105 degrees. In our study, the duration of surgeries ranges from three and seven hours.

**Table 1:** Demographic data of the patients and their procedures BMI: Body Mass Index, ASA: American Society of Anesthesiologists, C: Cervical, T: Thoracic, L: Lumbar, S: Sacral, H: Hours

Case no.	Gender	Age in years	BMI	ASA status	Cobb angle	Level of procedure	Procedure duration
1	Female	11	14.6	2	55 degrees	Posterior spinal fusion T4-L4	6 H
2	Female	8	14.1	2	51 degrees	Posterior spinal fusion T2-T3, L4-L5	5 H
3	Male	11	20.4	3	68 degrees	Posterior spinal fusion T2-T4, L4-S2	5 H
4	Male	8	11.9	2	55 degrees	Posterior spinal fusion T2-L3	3.30 H
5	Female	14	15.9	3	39 degrees	Posterior spinal fusion L4-S2	3 H
6	Female	14	15.23	2	44 degrees	Posterior spinal fusion C3-T3	3 H
7	Male	8	10.1	2	104 degrees	Posterior spinal fusion C4-L4	7 H

Table 2 details the time of first rescue medication and duration the patients were pain-free. In this study, there was only one case of nausea and vomiting, while the others had no adverse events such as respiratory depression, purities or haemodynamic changes. One patient experienced pain after 48 hours; however, one case had unspecific pain in two hours postoperatively, where he received morphine. Among seven cases we had no shivering effect immediately after surgery and in the post-anaesthesia care unit.

**Table 2:** Adverse events and the usage of analgesics on the patients H: Hours

Case no.	Nausea/vomiting	Respiratory distress	Shivering	Pruritus	Pain-free	Hypertension/hypotension	Time of first rescue medication
1	Nil	Nil	Nil	Nil	22 H	Nil	22 H
2	Nil	Nil	Nil	Nil	2 H	Nil	2 H
3	Nil	Nil	Nil	Nil	10 H	Nil	10 H
4	Once	Nil	Nil	Nil	28 H	Nil	28 H
5	Nil	Nil	Nil	Nil	24 H	Nil	24 H
6	Nil	Nil	Nil	Nil	48 H	Nil	48 H
7	Nil	Nil	Nil	Nil	24 H	Nil	24 H

## Discussion

Pain management for patients undergoing scoliosis surgery is a critical component of perioperative care aimed at enhancing recovery and minimising complications. Multimodal analgesia should be employed to manage pain effectively and reduce opioid requirements. This includes the use of intravenous patient-controlled analgesia or regional anaesthesia techniques such as epidural or intrathecal analgesia [7]. Meta-analyses studies show that epidural analgesia is effective analgesia for patients going for scoliosis correction compared to intravenous patient-controlled analgesia, leading to lower opioid requirement postoperatively [8,9].

In this study, the goal was to establish our experience with the usage of dexmedetomidine along with morphine in patients going for correction of scoliosis in the operating room and to determine the effects in the postoperative phase, such as analgesic effects and any adverse experiences, which were recorded after the usage of such medications.

Pain is considered the most important factor for many patients when undergoing scoliosis procedures; it can have deleterious mental and psychological effects, and make them less satisfied with the perioperative experience. As anaesthesiologists, our main goal is to aid in the perioperative delivery of medical care by improving pain management through individualised regimens for each patient and always finding new methods to improve the regimens to reach the utmost satisfaction of the patients.

Caudal morphine is known to have an effective analgesic effect that can last for less than 24 hours, with a low incidence of adverse events after pediatric cardiac surgery and a lower opioid requirement in the postoperative phase [10].

Dexmedetomidine, like many drugs in medicine, is considered a drug worth studying due to the benefits it has shown in multiple studies, specifically in pain management and reducing opioid analgesic requirements during the perioperative period also, Dexmedetomidine is a highly selective α2 agonist with sedative and analgesic properties; it inhibits the excitability of the sympathetic nerve and also inhibits the stress response [11]. The usage of dexmedetomidine in our study with a dose of 4 mcg was decided after discussion with a neurophysiology specialist to avoid any missing interpretation of neurophysiology signal during the procedure given that we found no study about caudal dexmedetomidine in patients going for scoliosis correction along with neurophysiology monitoring. Wang et al. (2020) concluded that the usage of dexmedetomidine prolonged postoperative analgesia and decreased intraoperative end-tidal sevoflurane concentration [12].

Dexmedetomidine can lower the risk of postoperative behavioural changes [13]. Also, it can be used safely by neuraxial mode for better satisfaction with pain management and for a better sedation effect [14,15]. Our study showed that first rescue medication was extended up to 48 hours in one patient; however, one patient required analgesia medication two hours after surgery. All seven cases had no episodes of either hypo- or hypertension in the operation room or postoperative period.

Limitations

There are still several limitations to our study. Firstly, it has a retrospective nature with a small number of patients that were included (Riyadh, Saudi Arabia). Secondly, no control group was added. Thirdly, the administration of dexmedetomidine and morphine was done by identifying the anatomical marking. For more precise results, ultrasound-guided administration would be more desirable and can lead to more accurate interpretation of outcomes.

## Conclusions

Scoliosis surgery is considered to be one of the most invasive surgical procedures that require pre-emptive and multimodal analgesia to enhance recovery. In this study, our objective was to present our experience with dexmedetomidine along with morphine as an efficient analgesic medication to be used intraoperative, as we found that it showed a high level of reliability in prolonging sedation and analgesia time, prolonging the usage of rescue medication and lowering any adverse events that may happen postoperatively. Further studies are needed to assess the use of neuraxial dexmedetomidine under neuromonitoring for patients going for scoliosis surgery.
